# Usefulness of intraoperative nerve monitoring for giant type AB thymoma combined with an aberrant right subclavian artery: a case report

**DOI:** 10.1186/s13019-022-02056-6

**Published:** 2022-12-08

**Authors:** Yoshihito Iijima, Masahito Ishikawa, Shun Iwai, Aika Yamagata, Nozomu Motono, Hiroyuki Tsuji, Hidetaka Uramoto

**Affiliations:** 1 Department of Thoracic Surgery, Kanazawa Medical University, 1-1 Daigaku, Uchinada-machi, Kahoku-gun, Ishikawa 920-0293 Japan; 2 Department of Head and Neck Surgery, Kanazawa Medical University, Kahoku-gun, Ishikawa Japan

**Keywords:** Thymoma, Surgery, Aberrant right subclavian artery, Nonrecurrent inferior laryngeal nerve, Intraoperative nerve monitoring

## Abstract

**Background:**

Abnormal tumor vascularization and escalating tumor size represent two major impediments that make cancer surgery impossible or complicated.

**Case presentation:**

Herein, we report the case of a giant thymoma (type AB) in a 58-year-old woman who presented with cough and yellow sputum. The thymoma grew extensively from the neck to the upper mediastinum. The patient exhibited an aberrant right subclavian artery and a non-recurrent inferior laryngeal nerve. Intraoperative nerve monitoring facilitated the identification and preservation of vital nerves spanning the neck and chest, including the non-recurrent inferior laryngeal nerve. Furthermore, the tumor was divided naturally along the constriction, and a good field of view was acquired to identify abnormal right subclavian arteries and nerves that ran deep in the tumor and surgical field. The tumor was safely removed without complications using intraoperative nerve monitoring, and the thymoma that grew extensively from the neck to the upper mediastinum and was associated with an aberrant right subclavian artery was resected.

**Conclusion:**

Intraoperative nerve monitoring was helpful in identifying the non-recurrent inferior laryngeal nerve and left recurrent laryngeal nerve.

**Supplementary Information:**

The online version contains supplementary material available at 10.1186/s13019-022-02056-6.

## Background

Particular tumor-associated conditions, such as excessive tumor size, malformations of surrounding organs, or aberrant blood vessels, can make it exceedingly complicated to surgically remove certain tumors. Here, we describe the successful resection of an enormous thymoma associated with major impediments to surgical intervention. The patient presented with an aberrant right subclavian artery (ARSCA), which is only found in 0.2–13.3% of the general population [1]. The anatomical nervous structure linked to a normal right subclavian artery (RSCA) connects the right recurrent laryngeal nerve with the RSCA. However, due to the abnormal running of the RSCA, the non-recurrent inferior laryngeal nerve (NRILN), which controls the laryngeal muscles, branches off at the neck. Therefore, many ARSCA cases are complicated by the presence of the NRILN [1, 2]. We successfully resected a massive thymoma exhibiting an ARSCA. The tumor location was extensive, spanning the neck and upper mediastinum, but excluded large blood vessels in the neck and chest.

## Case presentation

**Fig. 1:**
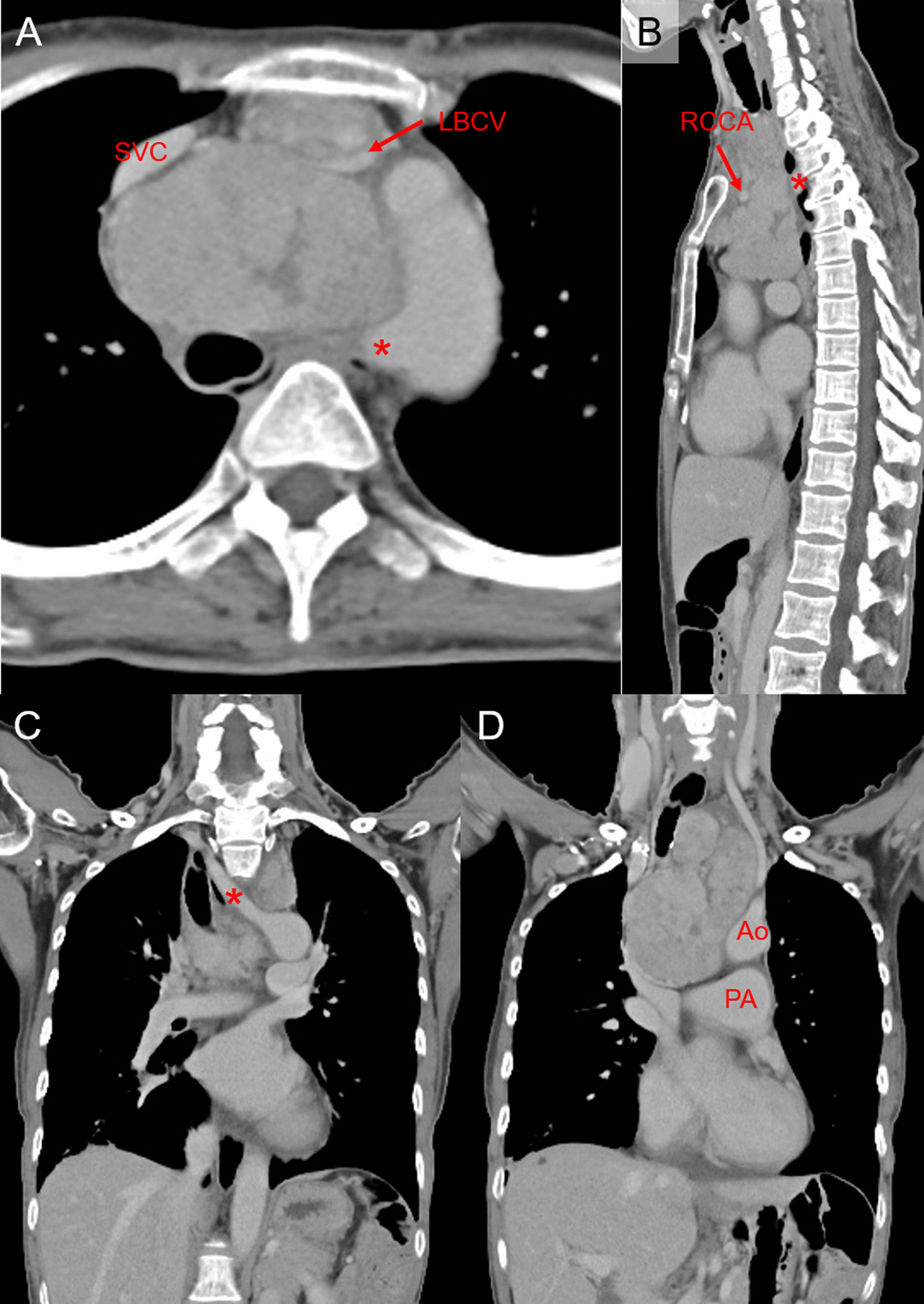
Chest contrast-enhanced computed tomography revealing a huge mass shadow with a nonuniform contrast effect inside, measuring 8.3 cm × 6.7 cm × 12.3 cm from the neck to the upper mediastinum. **A** Horizontal view, **B** sagittal view, and **C**, **D** coronal view. The red asterisk is aberrant right subclavian artery. *SVC* superior vena cava, *LBCV* left brachiocephalic vein, *RCCA* right common carotid artery, *Ao* aorta, *PA* pulmonary artery

**Fig. 2:**
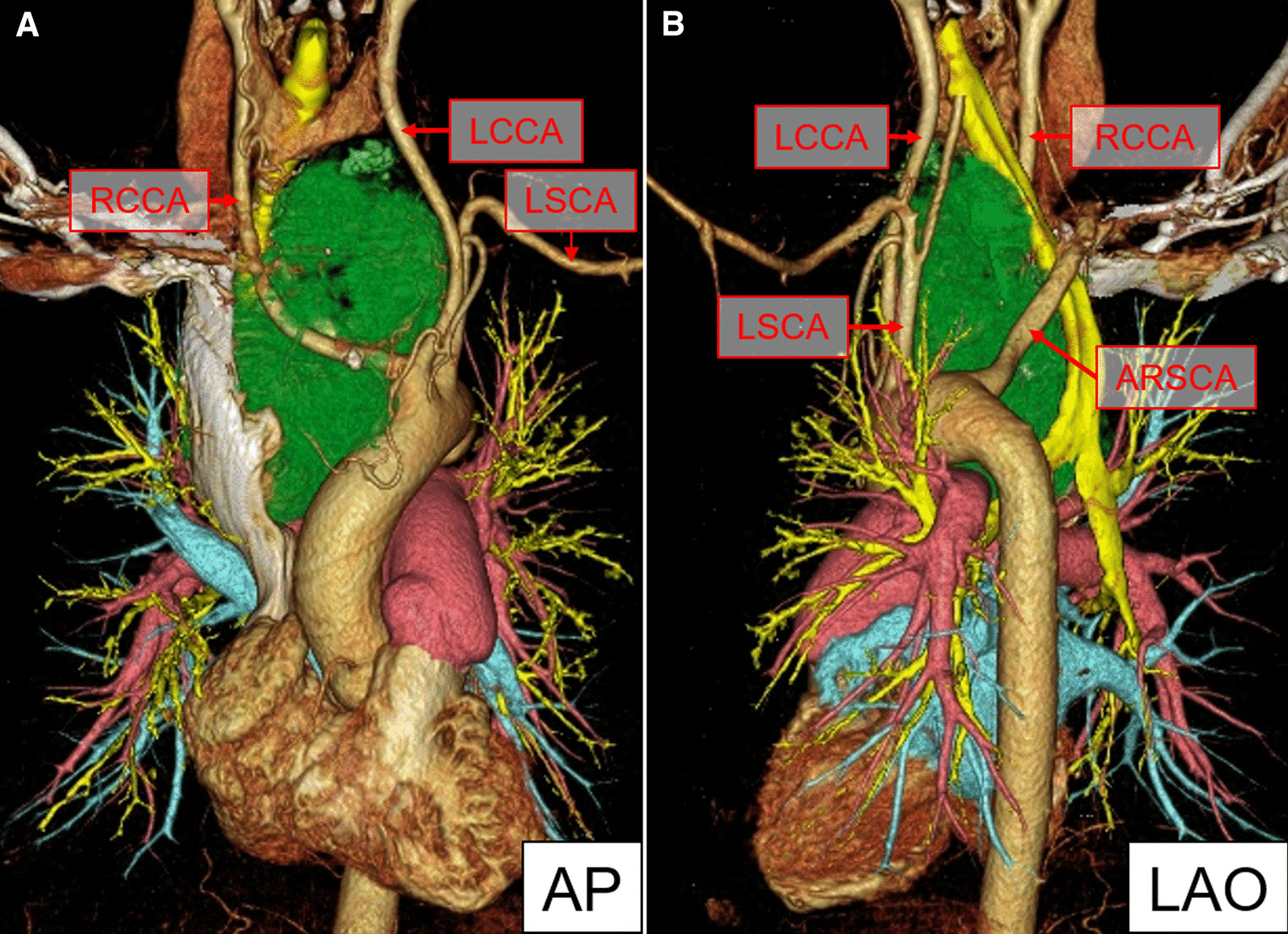
Three-dimensional angiography clearly demonstrating an aberrant right subclavian artery as the third branch of the aortic arch. **A** Anteroposterior view. **B** left anterior oblique view. *LCCA* left common carotid artery, *RCCA* right common carotid artery, *LSCA* left subclavian artery, *RSCA* right subclavian artery

A 58-year-old woman presented with cough and yellow sputum and was subsequently diagnosed with a neck tumor. Furthermore, an elastic hard mass was palpable in the neck. There was no swelling of the upper extremity or face. Chest radiography revealed that the trachea was displaced to the right, and an infiltration shadow was observed in the lower right lung field. In addition, the patient was diagnosed with *Mycobacterium gordonae* infection using a sputum culture test. A chest contrast-enhanced computed tomography (CT) scan revealed a huge mass shadow with a nonuniform contrast effect inside measuring 8.3 cm × 6.7 cm × 12.3 cm from the neck to the upper mediastinum (Fig. 1) as well as bronchiectasis with consolidation in the middle lung lobe. The aorta, superior vena cava, and pulmonary artery were excluded from the mass. The left brachiocephalic vein (LBCV) was stenotic due to compression by the mass from the ventral and dorsal sides, and tumor invasion was suspected. The boundary between the tumor and the superior vena cava was partially unclear, and invasion could not be ruled out. In addition, the common trunk of the left and right common carotid artery branches was the first branch of the aortic arch. The left subclavian artery constituted the second branch, and the RSCA the third branch. Moreover, the RSCA ran on the dorsal side of the tumor and trachea (Fig. 2). A biopsy was performed through the neck, revealing a T3N0M0 clinical stage III type A thymoma. Induction chemotherapy was not performed due to tumor-associated side effects leading to the deterioration of the patient’s general condition. A more decisive contraindication for chemotherapy induction was the risk of neglected surgery opportunities if chemotherapy proved ineffective. Thus, after evaluating of all therapeutic options, surgery was performed.

**Fig. 3:**
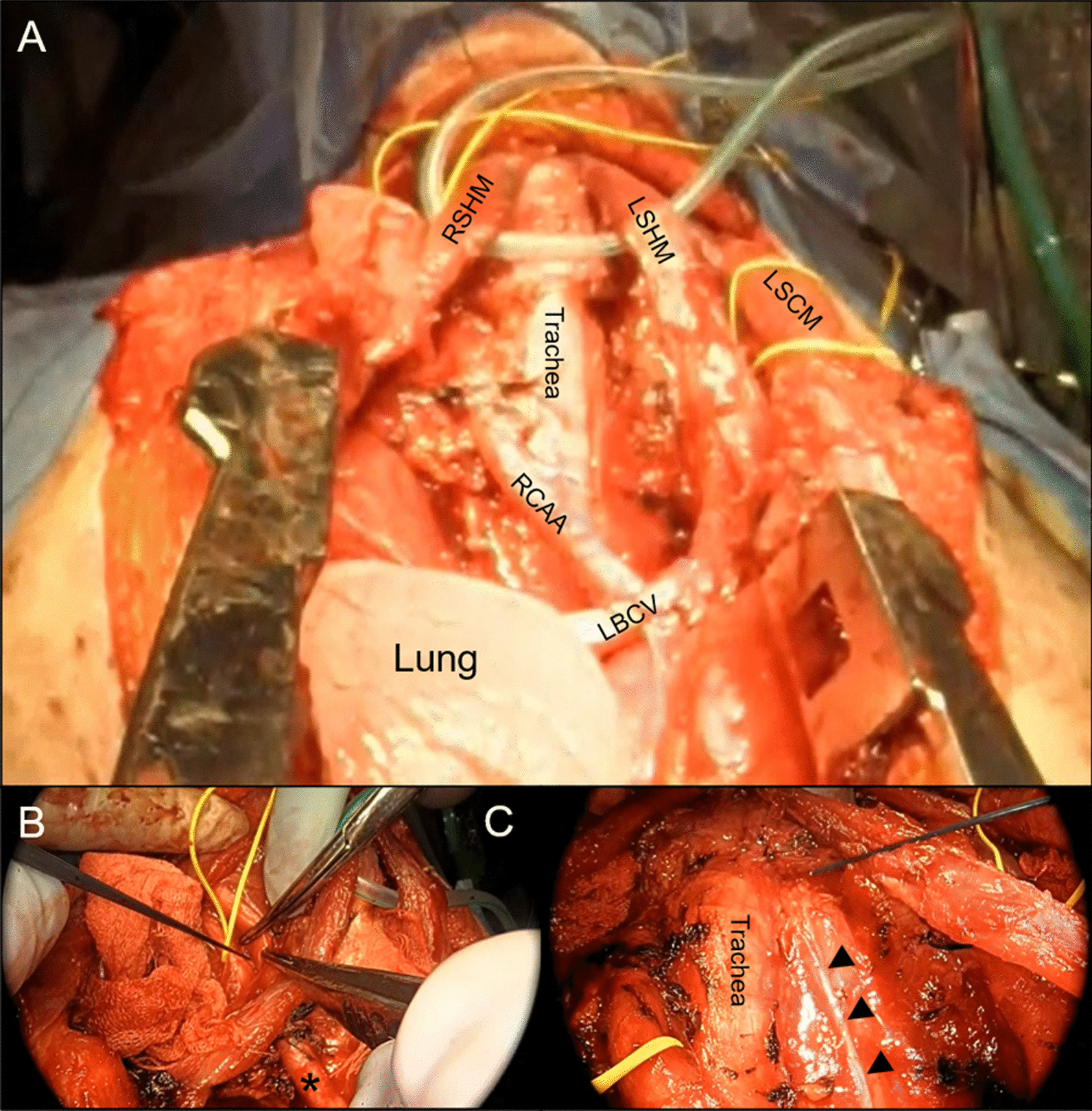
Intraoperative findings. **A** The tumor was removed without injury the surrounding organs. Both **B** the right non-recurrent inferior laryngeal nerve and **C** left recurrent laryngeal nerve were identified and preserved by intraoperative nerve monitoring using the impulses by the stimulation probe. The black asterisk indicates the RCCA and the black arrow-head indicates the left vagus nerve. *RCCA* right common carotid artery, *LSCM* left sternocleidomastoid muscle, *LBCV* left brachiocephalic vein, *RSHM* right sternohyoid muscle, *LSHM* left sternohyoid muscle

Thymothymectomy was performed using a cervical collar incision and median sternotomy (Fig. 3). During the cervical operation by the head and neck surgery team, the NRILN, left recurrent laryngeal nerve (LRLN), bilateral vagus nerves, and bilateral phrenic nerves were identified and preserved by intraoperative nerve monitoring (IONM) using the impulses from the stimulation probe. In addition, during the chest operation, the LBCV was only excluded from the anterior–posterior direction and was not infiltrated; the superior vena cava was found to be intact (Additional file 1).

**Fig. 4:**
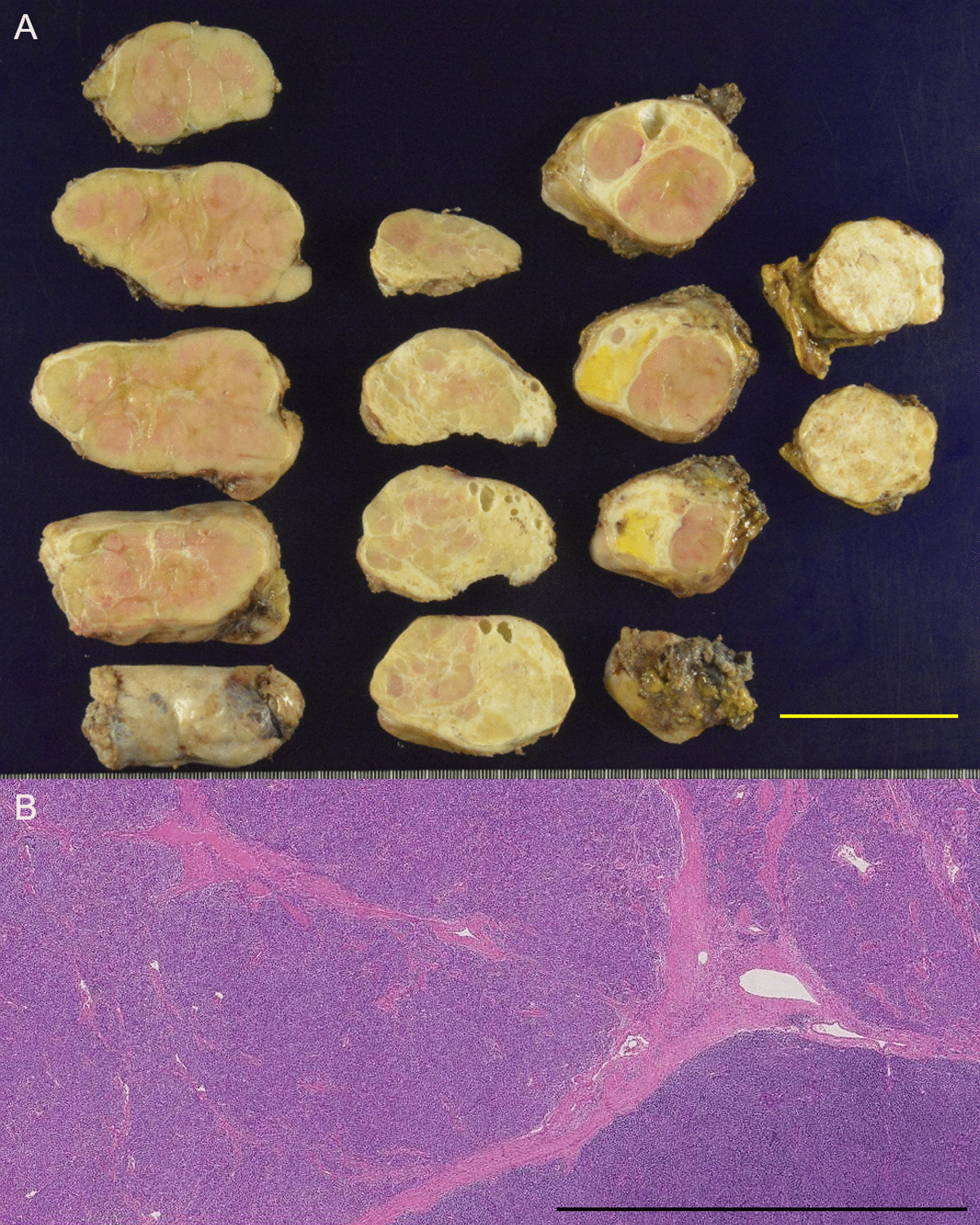
Pathological findings. **A** Macroscopic findings. The tumor was encapsulated with a fibrous tissue layer. The tumor is lobular due to the fibrous septum. The length of yarrow bar is 5.0 cm. **B** Histopathologically, the regions where spindle-shaped cells and rounded lymphocyte-like cells proliferated were mixed. Hematoxylin–Eosin stain, × 1.25 magnification. The length of black bar is 3.0 mm

The tumor was divided naturally along the constriction and was completely resected, and a good field of view was acquired to identify the ARSCA and LRLN that ran behind the tumor, deep in the surgical field. ARSCA exfoliation and LRLN preservation were achieved safely and reliably with this approach. Other operational circumstances were recorded as follows: the operation was performed in 305 min; the amount of bleeding was 160 mL; and all drainage tubes placed in the neck, substernal, and right thoracic cavities were removed by the third postoperative day. Histopathologically, the mass was determined to be a T1aN0M0 stage I type AB thymoma, and the tumor was encapsulated within a fibrous tissue layer (Fig. 4). Notably, no invasion was observed in the capsule. After the operation, the patient was discharged on the eighth postoperative day without complications, such as hoarseness, dysphagia, or phrenic nerve palsy. Bronchiectasis of the middle lobe of the right lung did not improve after surgery. The patient is currently an outpatient, three months after the operation.

## Discussion and conclusions

Whether to perform up-front surgery or surgery after induction chemotherapy was considered for the excision of this tumor. Following the preoperative biopsy, the patient was diagnosed with a type A thymoma, a colossal tumor with an ARSCA. Surgery was performed for several reasons; first, when considering the combined resection of the tumor and LBCV, it was judged that complete resection was possible. Second, steroid pulse therapy has an insignificant effect on type A thymomas [3]; however, there was concern about postoperative sternal dissection and mediastinitis due to steroids. Third, chemotherapy could make it difficult to control a potential postoperative infection due to bronchiectasis and the detection of nontuberculous mycobacteria in the sputum. Finally, we were afraid to miss the opportunity for surgery due to the onset of side effects associated with chemotherapy and tumor growth.

Since thymoma poses a risk of dissemination upon unintended damage to the capsule, the tumor should be resected as an en-bloc mass. However, in advanced-stage malignant tumors, the conflict between achieving oncologic R0 resection and patient safety remains an unsolved issue. The separation of tumors should also be considered under special circumstances [4–6]. In this case, preoperative evaluation of CT images showed that the border between the caudal side of the tumor and the superior vena cava was partially obscured, which may not result in complete resection. However, the tumor was divided naturally along the constriction when the LBCV that ran between the tumors was detached. A good field of view was acquired to identify the ARSCA and LRLN that ran deep in the tumor and surgical field; blood vessels could be safely detached with a clear visual field, and essential nerves could be preserved using IONM. While we believe that a complete resection was achieved, the tumor was unintentionally divided. Therefore, the risk of recurrence may have increased, and strict follow-up is required.

IONM assesses muscle-induced electromyography (EMG) by placing electrodes on the muscle and directly electrically stimulating the innervating nerve with a nerve stimulation probe during surgery to monitor nerve function [7]. It is a widespread,confirmatory method that has been recognized to be helpful in identifying and preserving recurrent laryngeal nerves in locally advanced thyroid cancer, reoperation cases, and giant goiter cases. Typically, when anesthesia is introduced, an EMG endotracheal tube is placed so that the electrode is in contact with the tracheal arytenoid muscle, making it is possible to detect the contraction of the cricothyroid muscle during recurrent laryngeal nerve stimulation. In this case, the EMG endotracheal tube could not be placed due to the size of the tumor, and an electrode was inserted into the patient's right shoulder for monitoring.

In conclusion, the thymoma that grew extensively from the neck to the upper mediastinum and was associated with an ARSCA, was resected. We were thus able to safely remove the tumor without complications. The use of IONM was useful in identifying the NRILN and LRLN.

## Supplementary Information

**Additional file 1.** Intraoperative findings. Thymothymectomy was performed using a cervical collar incision and median sternotomy. The nonrecurrent inferior laryngeal nerve, left recurrent laryngeal nerve, bilateral vagus nerves, and bilateral phrenic nerves were identified and preserved by intraoperative nerve monitoring. Aberrant right subclavian artery exfoliation was achieved safely and reliably with this approach.

## Data Availability

All data generated or analyzed during this study are included in this published article.

## Abbreviations

ARSCA: Aberrant right subclavian artery
RSCA: Right subclavian artery
CT: Computed tomography
EMG: Electromyography
IONM: Intraoperative nerve monitoring
LBCV: Left brachiocephalic vein
LRLN: Left recurrent laryngeal nerve
NRILN: Nonrecurrent inferior laryngeal nerve
